# Thymoquinone: A Tie-Breaker in SARS-CoV2-Infected Cancer Patients?

**DOI:** 10.3390/cells10020302

**Published:** 2021-02-02

**Authors:** Sawsan Elgohary, Aya A. Elkhodiry, Nada S. Amin, Ulrike Stein, Hend M. El Tayebi

**Affiliations:** 1 Molecular Pharmacology Research Group, Department of Pharmacology and Toxicology, Faculty of Pharmacy and Biotechnology, German University in Cairo, 11835 Cairo, Egypt; sawsanelgohary54@gmail.com (S.E.); ayakhod@gmail.com (A.A.E.); nada.sherif@guc.edu.eg (N.S.A.); 2 Experimental and Clinical Research Center, Charité—Universitätsmedizin Berlin, 10117 Berlin, Germany; ustein@mdc-berlin.de; 3 Max-Delbrück-Center for Molecular Medicine, 13125 Berlin, Germany; 4 German Cancer Consortium (DKTK), 69120 Heidelberg, Germany

**Keywords:** SARS-CoV-2, COVID-19, cancer, thymoquinone, apoptosis, COVID-19 treatments, anticancer drugs, COVID-19 complications, Nrf2, GRP78, RAS

## Abstract

Since the beginning of the SARS-CoV-2(severe acute respiratory syndrome-coronavirus-2) pandemic, a race to develop a vaccine has been initiated, considering the massive and rather significant economic and healthcare hits that this virus has caused. The pathophysiology occurring following COVID-19(coronavirus disease-2019) infection has given hints regarding the supportive and symptomatic treatments to establish for patients, as no specific anti-SARS-CoV-2 is available yet. Patient symptoms vary greatly and range from mild symptoms to severe fatal complications. Supportive treatments include antipyretics, antiviral therapies, different combinations of broad-spectrum antibiotics, hydroxychloroquine and plasma transfusion. Unfortunately, cancer patients are at higher risk of viral infection and more likely to develop serious complications due to their immunocompromised state, the fact that they are already administering multiple medications, as well as combined comorbidity compared to the general population. It may seem impossible to find a drug that possesses both potent antiviral and anticancer effects specifically against COVID-19 infection and its complications and the existing malignancy, respectively. Thymoquinone (TQ) is the most pharmacologically active ingredient in *Nigella sativa* seeds (black seeds); it is reported to have anticancer, anti-inflammatory and antioxidant effects in various settings. In this review, we will discuss the multiple effects of TQ specifically against COVID-19, its beneficial effects against COVID-19 pathophysiology and multiple-organ complications, its use as an adjuvant for supportive COVID-19 therapy and cancer therapy, and finally, its anticancer effects.

## 1. Introduction

In March 2020, the WHO declared coronavirus disease -2019 (COVID-19) as a pandemic; this served as the spark that ignited the global race for the development of a vaccine against the novel coronavirus, severe acute respiratory syndrome-coronavirus-2(SARS-CoV-2). This was evidently important amidst the sudden and substantial increase in hospitalizations for pneumonia associated with multi-organ disease. The pathophysiology of COVID-19 patients gave clues to suitable supportive and symptomatic treatment of this pandemic; however, no specific treatment against COVID-19 has been found to date. COVID-19 patients have many symptoms that vary in severity, including dry cough, fever [1], sore throat, fatigue, diarrhea, shortness of breath, myalgia, in addition to radiographic and laboratory or biochemical abnormalities [2]. Moreover, cardiovascular manifestations include. Acute cardiac injury, myocarditis, arrhythmia and cardiovascular thromboembolism havebeen frequently reported in COVID-19 patients [3]. Furthermore, dizziness, headache, taste and smell dysfunctions, or impaired consciousness has been frequently shown among neurological manifestations in COVID-19 patients [4,5]. Cases involving either viral co-infection or co-infection with both viral and bacterial pathogens that cause pneumonia have been described, particularly in the period following the initial phase of viral respiratory infection [6]. In severe cases, acute lung injury (ALI), acute respiratory distress syndrome (ARDS), respiratory failure, heart failure, sepsis, multiple organ dysfunction, and sudden cardiac arrest occur within a few days [7]. The supportive treatments include antipyretics, antiviral therapies, different combinations of broad-spectrum antibiotics, hydroxychloroquine and plasma transfusion [8]. Cancer patients are at higher risk (two-folds) for COVID-19 and are more likely to develop severe outcomes compared to the general population [9,10,11]. This is greatly attributed to immunosuppressive conditions, consumption of multiple medications, combined comorbidity, and are more likely to require ventilatory support or ICU admission [12,13,14,15]. They also have significantly higher fatality rates (28.6% compared to 2.3% for all COVID-19 patients in China) [12]. Patients who have received cancer treatment within 14 days of getting a COVID-19 diagnosis are considered to have a risk factor for developing adverse events, such as ARDS, septic shock, and acute myocardial infarction [12]. Despite this observation, delaying cancer treatment for patients also has well-documented complications [12]. It may seem rather challenging to discover a drug that shows not only antiviral effects specifically against SARS-CoV-2 infection and entry but also antimicrobial (antiviral, -bacterial, -fungal) that protects against SARS-CoV-2 co-infection induced pneumonia, ameliorate respiratory symptoms, as well as acts as an antioxidant thereby protecting against COVID-19 associated multiple organ dysfunction (i.e., cardio-protective, hepato-protective, gastro-protective and protects the kidney from injury). Moreover, this drug should also ideally have anti-inflammatory effects that are sufficient enough to ameliorate COVID-19 induced cytokine storm and whose overall pharmacological effects are consistent with the pathophysiology of COVID-19 in cancer patients.

TQ shows all the above-mentioned effects [16,17,18,19,20,21,22,23,24,25,26]. TQ is the most pharmacologically active ingredient in *Nigella sativa* seeds (black seeds) extract [27,28]. In this review, we will be discussing the potential effects of TQ as a SARS-CoV-2 antiviral drug, its beneficial effects against COVID-19 pathophysiology with a focus on cancer patients, as well as some of its anticancer effects and its use as an adjuvant besides supportive COVID-19 therapy and cancer therapy. To achieve the purpose ofthe review, research was conducted at the States National Library of Medicine (PubMed). For the search in databases, the descriptors used were: “thymoquinone” and “COVID-19”/“SARS-CoV-2” or “cancer”, “COVID-19”/“SARS-CoV-2” and “cancer” or “apoptosis”, “thymoquinone” and various COVID-19 complications and key molecular pathways. Research papers, books, and published data were reviewed for their relevance to the aim of the review and summarized. Criteria for inclusion were complete, relevant publication, available online, in English, published between 2010 and 2020, with detailed information about participants, methods, and analyses. Data collection was done during August 2020, and data abstracted was in the form of descriptive information, covering the type of samples used, techniques, and findings or effects reported. Bias was limited through the evaluation of the studies through their internal validity rather than the conclusion.

## 2. Sars-Cov-2 Structural Features Involved in Entry

Understanding the structural features of SARS-CoV-2 and its replication is pivotal to focus on molecular targets lessening the viral entry, replication and to fully understand the possible treatment options. SARS-CoV-2 belongs to the family of beta-corona viruses (β-CoVs) [29] that have the crown-shaped spike (S) trans-membrane glycoprotein. The human serine proteases participate in viral attachment and RNA entry into the cell. The transmembrane serine protease 9 (TMPRSS2) and endogenous serine proteases, such as furin, prime and cleave the S spike into S1 and S2; this separation is essential for the attachment of the SARS-CoV-2 to both the angiotensin-converting enzyme 2 (ACE2) receptor (S1-part of spike) and the cell membrane (S2-part of spike). The attachment of the viral spikes to both the ACE2 receptor and the cell membrane is necessary for successful viral entry and the delivery of its RNA into the cytoplasm [30,31], where it is ready to be translated as mRNA.

Since viral spike attachment to ACE2 receptors is key for its entry and infection of cells, the location of these particular receptors explains a lot about SARS-CoV-2 entry and subsequent COVID-19 symptoms. ACE2 receptors are highly expressed in the lungs (for example, on type 2 alveolar cells (AT2)), nose, oropharynx, kidney proximal tubule cells, bladder urothelial cells, cells of the gastrointestinal tract [12] and myocardial cells [32,33]. This explains why COVID-19 patients do not only experience respiratory problems like pneumonia that might lead to ARDS but may also experience heart, kidneys and digestive tract disorders. The susceptibility and vulnerability of cancer patients to COVID-19 is a multi-factorial issue. Cancer patients tend to have higher levels of ACE2 expression; they are therefore more likely to experience more adverse outcomes [13]. Additionally, TMPRSS and cathepsin L (CTSL), two key enzymes that form essential interactions with the viral spike proteins, were found to be upregulated in certain types of cancer, as shown in Table 1 [34].

## 3. Benefits of Thymoquinone as an Adjuvant in COVID-19

As mentioned previously, there is no targeted therapy for SARS-CoV-2; supportive treatments against the disease manifestations are undertaken as reviewed in [35,36]. Supportive therapy includes steroids to alleviate the inflammatory status; literature showed that TQ improved oxygenation when combined with steroids and showed protective effects in the lungs [37]. Non-steroidal anti-inflammatory drugs like diclofenac are also used to counteract COVID-19-induced fever and myalgia [38]. Furthermore, COVID-19 and cancer patients are at high-risk of acetaminophen-induced hepatotoxicity as reviewed in [39], diclofenac-induced gastrointestinal side effects as reviewed in [40], along with renal toxicity [41]; luckily, TQ administration protects against acetaminophen-induced hepatotoxicity [42]. In a recent study, TQ protected against diclofenac-induced kidney injury [42,43] and ameliorated GIT toxicity with proton pump inhibition activity [44,45]. Moreover, TQ counteracted the toxic effects of other supportive therapies like gentamicin [46,47], chloroquine [48] and vancomycin [25] and showed a synergistic effect when administered with ranitidine [44,45]. Furthermore, tocilizumab, an IL-6 receptor antagonist, has been used in various cancers, such as multiple myeloma and solid tumors, including renal, prostate, lung, colorectal and ovarian cancers, to decrease cancer-associated inflammation. It is used alone or in combination with conventional chemotherapy, as reviewed in [49]. In COVID-19 patients, tocilizumab is given to decrease the viral-induced IL-6 elevation, cytokine storm, and inflammation [50]. TQ is reported as a potent anti-inflammatory drug that decreases IL-6 expression and inhibits NFĸB [51]. Hence, giving TQ with tocilizumab could give double beneficial effects in COVID-19 patients. It has been reported that even a single dose of tocilizumab caused gut ulceration in COVID-19 patients [52], and since TQ showed gastroprotective effects, it would be beneficial to investigate the GIT protective effects of TQ in combination with tocilizumab against gut ulceration. Table 2 shows details about the use of TQ as a potential adjuvant therapy to minimize COVID-19 supportive treatments’ side effects.

Thus, far, this literature review has attempted to highlight how TQ could represent a potential adjuvant molecule to COVID-19 standard supportive treatments. To create a full picture, and in order to visualize the true value of TQ in COVID-19 cancer patients, the potential anticancer effects of TQ will be reviewed in the next section, as well as its potential benefit as an adjuvant to chemotherapy.

## 4. Anticancer Effects of Thymoquinone

For more than 50 years, TQ has been known for its anti-neoplastic and chemo-preventive effects that have been proven in various types of cancer [55]. Even though TQ is not an FDA-approved drug for cancer therapy, several in vitro and in vivo studies showed that the anticancer activity of TQ is mediated through different pathways involved in proliferation, cell cycle regulation of apoptosis, angiogenesis and cancer metastasis. Apoptosis or programmed cell death is considered to be the most powerful shield against cancer, as it maintains body homeostasis by eliminating aged or diseased cells from the body [56]. This is why mutations or dysregulations in genes regulating apoptosis, such as members of the Bcl-2 family, caspases, p53 and PTEN, are usually present in human cancers [57,58,59,60]. The antiproliferative and apoptotic effects of TQ are modulated through several mediators and transcription factors, along with its anti-angiogenic, antimetastatic and chemo, radio-sensitizing effects.

*TQ and apoptosis:* the downregulation of the antiapoptotic proteins Bcl-2, Bcl-xl, XIAP, survivin, and the upregulation of proapoptotic proteins Bax, p21 and p53 have been associated with TQ-induced apoptosis in various cancers [61,62,63]. TQ upregulating effect on the proapoptotic transcription factor, p53, was clearly emphasized in numerous in vitro and in vivo studies on cancer models, where TQ induced apoptosis through the upregulation of p53 in breast cancer [64,65,66], renal cell carcinoma [67], leukemia [68], glioblastoma [69,70], squamous cell carcinoma [71], cervical cancer [72], lung cancer [73], and osteosarcoma [74]. The downstream protein of p53, p21 [75,76,77], was shown to be upregulated in pancreatic ductal adenocarcinoma as well [78]. The diminished expression of Bcl-2, Bcl-xl, survivin, c-FLIP and XIAP following TQ treatment has been established in several cell lines and animal models [79,80,81,82,83,84]. This diminished expression of Bcl-2 is usually coupled to increased Bax expression as reported in liver cancer [85], leukemia [83], bladder cancer [84], squamous cell carcinoma [86], ovarian cancer [82], and renal cancer [87]. Consequently, the disruption in the Bcl-2/Baxratio, accompanied by reactive oxygen species (ROS) generation induced by TQ inside cancer cells, damages mitochondrial membrane potential, leading to the release of cytochrome c. This triggers the intrinsic pathway and activates procaspases 9, 7, 3 and PARP enzyme cleavage. This cascade of events is seen in leukemia/lymphoma [88,89], glioblastoma [79,90], renal cell carcinoma [91], colon [92], brain [93], bladder [84] and ovarian cancers [82]. TQ has been reported to exert a dual effect in which it acts as both pro-oxidant and antioxidant in a dose-dependent manner; the antioxidant effect occurs at low concentration (<5 μM) whereas, at higher concentrations (>20 μM), it possesses pro-oxidant property [94]. TQ-induced cell death was mediated through caspase-3 activation in glioblastoma [79], liver [95,96] and cervical cancer [72], while caspases 9 and 3 mediated apoptosis wasseen in lymphoma [88], lung [73], and liver cancer [97]. Moreover, the induction of apoptosis in leukemia was mediated by caspases 8 and 3 involving more than one apoptotic pathway [83].

*TQ and cell cycle arrest:* since microtubule assembly is important in cell cycle progression, microtubule targeting is a potential mechanism for chemotherapy. TQ was reported to induce cell cycle arrest through depolymerizing microtubule network and disrupting the mitotic spindle organization in A549 lung cancer cells [98]. Several studies reported TQ’s cell cycle arrest action through inhibition of cyclin D, cyclin E and cyclin-dependent kinase 2 (Cdk-2) in various cancers [77,92,99,100]. Additionally, a death receptor-mediated-apoptosis was seen with TQ treatment, as TQ caused re-localization of CD95/Fas from cytoplasm to the membrane, enhancing apoptosis in multiple myeloma [101] and enhancing TRAIL/TRAILR expression in medulloblastoma [93], liver [96,97] and lung cancers [76].

*TQ* vs. *carcinogen activation:* One of TQ’s anticancer mechanisms is the suppression of carcinogen activation through inhibiting metabolizing enzymes that are responsible for the biotransformation process of various pro-carcinogens into highly active carcinogens. These reactive carcinogens lead to oxidative damage, or covalent modification of DNA, as well as changes in signaling networks [102,103,104]. Of particular interest in the cytochrome p450 family are CYP1 members, specifically CYP1A1 phase I enzyme, that was found to be subject to inhibition by TQ, accompanied byan elevation in glutathione and phase II GST enzyme in HepG2 cells [85]. Another anticancer mechanism lies in the anti-inflammatory effects of TQ through inhibiting or modulating cyclooxygenase-2 (COX-2) enzyme, which is known to catalyze the formation of the proinflammatory mediators, prostaglandins (PGs). TQ was reported to inhibit COX-2 and PGE in colorectal cancer [105] and cholangiocarcinoma cell lines [80].

*TQ* vs. *angiogenesis and metastasis:* TQ is thought to inhibit tumor angiogenesis by inhibiting the expression of vascular endothelial growth factor (VEGF) and its downstream signaling on cholangiocarcinoma [80], stomach [106] and triple-negative breast cancer (TNBC) cells [107]. Moreover, TQ is known to inhibit tumor migration, invasion and consequently tumor metastasis through modulating genes, transcription factors, and proteins responsible for facilitating the migration process [108]. A study on TNBC concluded that TQ exhibited its effect through inhibiting eEF-2K-signaling pathway with its downstream targets, Src kinase, focal adhesion kinase (FAK) and AKT following NF-kB inhibition [109]. Other studies reported that the antimetastatic effect of TQ is exhibited through the inhibition of PI3K/AKT/mTOR pathways, leading to the reversal of endothelial mesenchymal transformation (EndMT). This results in the upregulation of endothelial markers E-cadherin and cytokeratin-19, and downregulation of mesenchymal markers N-cadherin, vimentin, Twist, Slug, Snail, ZEB1 in renal cell carcinoma [110], gastric [111], prostate [112], cervical [113,114], bladder [115] and breast cancer [107,116]. A study on renal cancer cells concluded that the antimetastatic effect of TQ, facilitated by autophagy, is regulated by the AMPK/mTOR pathway [117]. Furthermore, the anti-migratory action of TQ is also exerted by the inhibition of MMP-2, MMP-9 along with integrin B1, urokinase-type plasminogen activator (u-PA), fibronectin, RhoA, and suppression of the activation of JNK, p38, EGFR/IKKa/b/NF-kB-signaling, paxillin and Gαi2. This was observed in cancers like TNBC [107], lymphoma [118], glioblastoma [119], renal cell carcinoma [120], colon [121,122] and ovarian cancers [123]. It was also reported that TQ causes downregulation in the TGF-β/Smad2/3-signaling pathway and reduction in CXCR4/CXCL12 pathway mediated by NF-ĸB expression [112,124,125]. Finally, TQ caused inhibition in Wnt/B-catenin signaling with a consequent reduction in β-catenin target genes, Myc, Axin-2, MMP-7, cyclin D1 and Met in bladder cancer cells [126], and inhibiting ERK1/2 phosphorylation in glioblastoma [127] and lung cancer [128].

**Figure 1:**
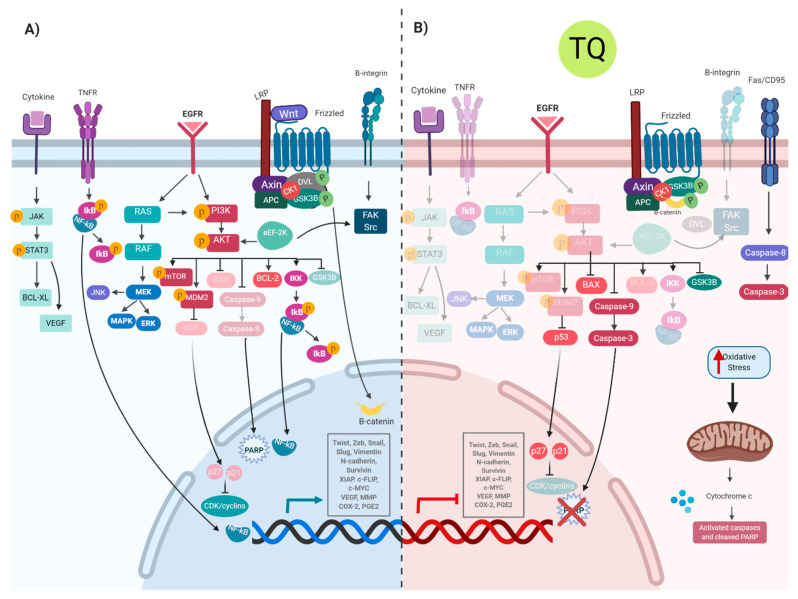
**Molecular mechanisms of TQ in cancer development** (**A**) pathways involved in cancer development; (**B**) pathways inhibited by TQ that appear transparent in the figure while opaque molecules are activated.

*TQ* vs. *gene transcription:* the effects of TQ are related and can possibly be linked together by the transcription factors involved. TQ treatment inhibits signal transducer and activator of transcription-3 (STAT3) phosphorylation, causing downregulation of STAT3 and its related genes, Janus activated kinase-2 (JAK2), c-Src, Bcl-2, cyclin D, survivin, VEGF and caspases 3, 7 and 9 in gastric cancer [129]. The downregulation of the PI3K/Akt pathway and NF-ĸB leads to changes in various related genes, NF-ĸB p65, GSK3B, XIAP, Bcl-2, VEGF, COX-2, PGE2, inducing apoptosis and inhibiting metastasis in cancer cells [130,131,132]. Another TQ-affected signaling pathway is the tumor necrosis factor (TNF) pathway, which usually induces NF-ĸB-signaling, hence modulating apoptosis and inflammation. This pathway was found to be modulated following the treatment of HeLa cells with TQ [133]. Following NF-ĸB inhibition, NLRP3 inflammasome is inhibited, causing inactivation of caspase-1 and inhibition of IL-1 and IL-18 in melanoma cells [134]. Moreover, TQ binds to PAK1, causing conformational changes, affecting RAF/MEK/ERK1/2-signaling pathway [105]. Figure 1 summarizes the signaling pathways contributing to carcinogenesis and how TQ inhibits them through its anticancer mechanisms.

*TQ as a chemo-adjuvant:* adding to the previously mentioned anticancer effects of TQ, several studies reported the potentiating effect of combining TQ with chemotherapeutic agents and radiation. It was shown to enhance their anticancer activity, causing chemo- or radio-sensitization, respectively [135,136,137]. It also reduced or attenuated some treatment-induced toxicities, such as nephropathy or hepatotoxicity, seen with cisplatin treatment [138,139,140,141]. Table 3 discusses some of the chemo-modulating effects of TQ in combination with known anticancer drugs. However, as with any other drug, further studies should be done to investigate the standardization of TQ administration when used with cancer drugs, as literature showed that CYP1A2, CYP2C9, CYP2D6 and CYP3A4 were subject to inhibition by TQ, hinting at various potential drug–drug interactions [142].

## 5. Thymoquinone’s Double Hits in COVID-19 Infected Cancer Patients

First of all, a recent study showed that TQ could work as an antiviral against SARS-CoV-2 since it revealed that TQ mighthave inhibitory activities against its viral protease in silico [161], and several other studies are also working on proving the same effect through insilico computational analysis. This rather interesting observation prompted the analysis of TQ’s reported roles in regulating proteins that are involved in COVID-19 as well as in cancer pathogenesis. This review, therefore, aims to summarize in the following sections the common protein targets that are subject to regulation by TQ and whose roles are furthermore crucial in COVID-19 cancer patients.

***Heat shock protein A5 (HSPA5)/GRP78*** is also known as immunoglobulin heavy chain-binding protein (BiP) or glucose-regulated protein (GRP78). GRP78 overexpression can occur under a variety of stressful conditions. This overexpression can cause the protein to be, in turn, highly abundant on the cell surface, where it can further potentiate the stressful conditions under which its expression was increased. For instance, overexpression of GRP78 on the cell membranes can increase viral entry via recognition and binding to the substrate-binding domain (SBD) of GRP78. This is true for several viruses [162], and molecular docking also showed that SARS-CoV-2 spike protein could bind to the host cell surface GRP78 [163]. Furthermore, the literature confirmed the in vitro presence of GRP78 protein in airway epithelial cells and in situ protein expression of GRP78 in the respiratory mucosa [164]. Higher serum GRP78 concentrations were found in COVID-19 patients compared to patients with pneumonia and the control group [165]. In another example, GRP78 can be overexpressed in the setting of a malignancy, where its abundance on cancer cell membranes endows antiapoptotic properties to the tumor cell by increasing the levels of antiapoptotic proteins such as Bcl-2 and reducing the levels of proapoptotic proteins, such as Bax. Ultimately, this results in promoting tumor survival, progression, angiogenesis, invasion, metastasis, as well as resistance to therapy [166]. This has been observed in various types of cancers [167,168,169,170,171].

**Figure 2:**
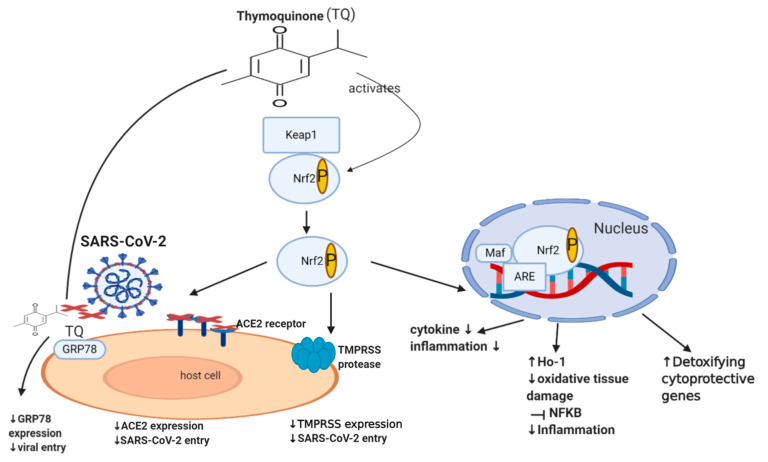
**Effect of TQ on Nrf2, GRP78 and SARS-CoV-2 infection**. A diagram showing the effect of TQ on Nrf2; it activates Nrf2 by phosphorylation, causing its translocation inside the nucleus, binding to ARE (antioxidant response element) and Maf. This binding results in the reduction of NF-kB, cytokine production, inflammation, oxidative damage and an increase in detoxifying cytoprotective genes and enzymes such as the HO-1 enzyme. Moreover, TQ decreases GRP78 expression, angiotensin-converting enzyme 2 (ACE)-receptor expression and hence decreases viral entry.

*HSPA5/GRP78 and TQ:* TQ decreases the expression of HSPA5/GRP78 and improved mitochondrial function [172]. Furthermore, molecular docking showed that TQ might interfere with SARS-CoV-2 attachment to HSPA5 by tightly binding to this protein on the cell surface [173]. Collectively, TQ produces a dual hit, where it tightly binds to HSPA5/GRP78, reducing its expression. Thus both reduces the risk of SARS-CoV-2 infection and decrease chemotherapy resistance, cancer invasion, metastasis, and survival, as illustrated briefly in Figure 2 [173].

***Nrf2 (nuclear factor erythroid-derived 2 related factor 2)*** is an important transcription factor that counteracts oxidative stress, where it acts as a sensor of oxidative stress, preventing genomic instability. It regulates about 250 genes involved in cellular homeostasis, including detoxifying enzymes, antioxidant proteins, and cytoprotective proteins [174]. Under normal physiological conditions, Nrf2 is sequestered by the cytoplasmic keap1 (Kelch-like ECH-associated protein 1), which maintains the Nrf2 at low levels through targeting it for proteasomal degradation (ubiquitination) [175]. Disruption of Nrf2 homeostasis can be seen in various settings, including viral infection and cancer. During a viral infection, intracellular expression of viral proteins leads to an increase in the oxidative stress of a cell. This leads to the dissociation of Nrf2 from keap1, which consequently prevents its ubiquitination [176,177]. Nrf2 is now able to translocate to the nucleus and activate the transcription of detoxifying, cytoprotective genes such as heme oxygenase (HO-1) [178,179]. This mechanism is also involved in the setting of cancer, where Nrf2 is additionally able to protect a cell from chemical and radiation-induced carcinogenesis [180,181,182,183]. Moreover, literature revealed that Nrf2 is able to enhance innate immune system activity, as well as participate in the inhibition of inflammatory cytokine expression, including IL-1β, IL-6, and NF-ĸB, ultimately decreasing inflammation [184,185,186]. Although some studies show that the continuous activation of Nrf2, as a result of excessive levels of ROS, could have deleterious effects on the host cell [187,188], it usually does not happen in viral infections since the virus needs to keep optimal oxidative stress levels allowing it to maintain viral metabolism without causing death in the host cell [189]. A recent study in 2020 involving 40 patients showed that the severity of COVID-19 is inversely associated with Nrf2 expression and directly linked to age and intensity of inflammatory response [190]. Interestingly, recent studies showed that Nrf2 deficiency upregulates ACE2 receptors, while activation of Nrf2 downregulates ACE2 receptors, with Nrf2 knock-out mice showing enhanced ACE2 expression. In cultured immortalized renal proximal tubule cells, treatment with Nrf2 inhibitor (Trigonelline) or transfection with Nrf2 small interfering RNA led to an increase in ACE2 transcription [191]. The exact mechanism of how Nrf2 downregulates the ACE2 receptor remains unclear. Additionally, Nrf2 activators downregulate the mRNA expression of TMPRSS2 [192,193] via upregulating TMPRSS2 inhibitors PAI-1 plasminogen activator inhibitor-1 encoded by the SERPINE gene (SERPINE/PAI-1) [192] and secretory leukocyte protease inhibitor (SLPI) [193]. This may highlight the important role of Nrf2 in downregulating TMPRSS2, ACE2 receptor expression and subsequently decreasing SARS-CoV-2 infection load. Despite the numerous cytoprotective mechanisms of Nrf2, it still appears to be a double-edged molecule since there is cumulative evidence establishing the fact that Nrf2 is one of the pathways that drive cancer progression, spread or metastasis, and chemo-resistance [194,195,196,197,198,199,200,201,202,203], and hence further dedicated studies may still be required.

*Nrf2 and TQ*: in keratinocytes, TQ works as a pro-oxidant that activates Nrf2 and increases its nuclear accumulation, leading to increased mRNA and protein expression of HO-1 [204], enhancing its binding to ARE, thus decreasing ACE2 expression, as demonstrated in Figure 2 [205]. During the course of COVID-19, white blood cells become activated, releasing excessive, prolonged inflammatory cytokines, leading to what is called the cytokine storm, acute respiratory distress syndrome, multiple organ dysfunction, and even death [206,207]. In addition, SARS-CoV-2 might invade the central nervous system inducing neurological diseases [208] manifested as headache, nausea, and vomiting. TQ showed inhibitory effects on NF-ĸB mediated neuroinflammation, as mentioned in Table 4, reporting other TQ effects in COVID-19-induced neurological disease. Altogether, TQ activates Nrf2 [204] that decreases ACE2 expression [205], potentially decreasing SARS-CoV-2 infection, inhibiting NF-ĸB [205], thus ameliorating the COVID-19-associated cytokine storm and inflammation. This would indeed also be beneficial in cancer patients who suffer from elevated cytokines and inflammation. On the other hand, Nrf2 appears to have contrasting roles in cancer patients, where it protects against chemical and radiation-induced carcinogenesis [180,181,182] but also leads to tumor-promoting effects. It would be beneficial to investigate the effects of TQ on Nrf2/HO-1/NF-ĸB in cancer patients with COVID-19.

***The Renin-Angiotensin System (RAS)*** is a homeostatic loop that begins when the hepatic angiotensinogen is converted into angiotensin I (ATI) by the renal renin enzyme. This loop then involves two arms; the ACE enzyme (from the lungs) converts ATI to angiotensin II (ATII), increasing its circulating levels. This mediator is implicated in vasoconstriction, fibrosis, hypertension, and inflammation. The second arm involves the conversion of ATII to AT1-7 by ACE2, which carries out the opposite effects of its precursor, ATII [242,243]. Many of the cardiovascular symptoms, as well as multiple organ damage seen with COVID-19, can be linked to RAS-induced dysregulation [244]. In fact, patients with COVID-19 tend to have significantly higher levels of circulating ATII compared to healthy individuals, and the findings of various studies that were conducted on autopsies [245] suggest that SARS-CoV-2 may cause downregulation of ACE2 receptors. This finding, along with receptor internalization upon viral binding, further contribute to the increase in ATII levels and the imbalance between the pro- and anti-inflammatory roles of the RAS, ultimately causing the proinflammatory role of the RAS to predominate [13], endowing procoagulant properties to endothelial cells [245].

The increased ATII enhances the activity of NADPH oxidase, increasing ROS, leading to induction of vascular smooth muscle cell (VSMC) proliferation, migration, atherosclerosis [246], and hypertension via increased oxidative stress, inflammatory reactions and production of vasoconstrictors while decreasing the vasodilator nitric oxide (NO). Thus, inhibition of VSMC proliferation and migration would be beneficial in preventing atherosclerosis and avoiding cardiovascular complications [247]. Peroxisome proliferator-activated receptor γ-coactivator 1α (PGC-1 α) was shown to attenuate ROS generation, thereby limiting ATII-induced rat VSMC proliferation [248]. Moreover, AMPK activation attenuates ATII induced VSMC proliferation, and PPARγ has been reported to have antiproliferative effects in multiple cancers [249,250]. The ramifications of the prolonged basal inflammatory state observed in the setting of malignancy can be closely mirrored in other statesof inflammation, including hypertension, obesity, diabetes, and even in COVID-19 patients with the aforementioned comorbidities. This surge in inflammatory cytokines increases the production of local and systemic ATII [251]. Increased levels of ATII in cancer patients has been associated with shortened survival [252]. It is therefore hypothesized that two hits contribute to the hyper-inflammatory response that is observed in COVID-19 patients with comorbidities; the first hit was resulting from the chronic state of inflammation, which increases the activity of the ATII proinflammatory axis, and the second hit resulting from SARS-CoV-2 itself, via the internalization of ACE2 receptors, and the decrease in the activity of the anti-inflammatory ACE2 axis [253]. Together, these two hits increase tissue damage as well as the risk for multiple organ failure.

*TQ and RAS/ATII*: the effect of TQ on ATII can be seen when TQ (40 mg/kg) is injected intraperitoneally 30 min before ATII (300 mg/kg) intravenous injection. This significantly blunted the rise in systolic blood pressure, mean arterial pressure and heart rate that wasinduced by ATII. Thus, the antagonistic effect of TQ on ATII appears to be beneficial in both COVID-19 patients and cancer patients [254]. TQ markedly inhibited ATII-induced VSMC proliferation, migration, and oxidative stress. It also reversed the elevated NADPH oxidase activity and ROS induced by ATII [255]. In addition, TQ enhanced the expression of p-AMPK, PPARγ, and PGC-1 α that were inhibited by ATII. These particular effects of TQ were abolished by AMPK inhibitor or PGC-1 α siRNA transfection [255]. Furthermore, TQ reduced oxidative stress, lipid peroxidation (specifically renal MDA), renal TNF-α in unilateral ureteral obstruction (UUO), and increased antioxidant enzymes (SOD, catalase). Meanwhile, it significantly decreased obstruction-induced apoptotic cell death and decreased ATII renal expression [256]. Thus, collectively, TQ improves renal oxidative damage, apoptotic cell death, TNF-α expression and also prevents the upregulation of ATII in UUO injured rats [257]. Looking at the bigger picture, TQ can be a promising drug in ameliorating COVID-19-induced RAS dysregulation, counteracting multiple organ failure in COVID-19 cancer patients.

## 6. The Significance of Thymoquinone in Other Pathological Processes in COVID-Cancer Patients

*Platelet hyper-reactivity*, the novel coronavirus, promotes a state of hypercoagulation [13] and additionally predisposes some patients to thromboembolic events through altering platelet function. Literature showed that resting platelets from COVID-19 patients had increased basal *P*-selectin expression, aggregated faster, and showed increased spreading on both fibrinogen and collagen. The increase in platelet activation and aggregation could partially be attributed to increased MAPK pathway activation and thromboxane generation [258,259]. Interestingly enough, TQ is able to ameliorate platelet hyper-reactivity by suppressing COVID-19-induced MAPK activation, and another study showed that TQ triggers apoptosis of blood platelets in a PI3K-dependent manner. Hypercoagulation in COVID-19 is typically characterized by elevated levels of fibrinogen and D-dimer, increased prothrombin time (PT), activated partial thromboplastin time, mild thrombocytopenia, elevated factor 8 and Von Willebrand Factor [12]. D-dimer levels are especially increased in critically ill patients who are administered into the ICU and is used as a prognostic factor [13]. Evidence suggests that SARS-CoV-2-induced release of proinflammatory cytokines of the innate immunity, either in close proximity to the affected organ (alveolar cells of the lung) or in the bloodstream, represents the culprit of the inflammatory process [12,206]. Concerning cancer patients, studies showed that multiple myeloma (MM) patients have an increased risk for venous thromboembolism (VTE) [258,259]. In this regard, TQ has been shown to reverse cancer-associated thrombosis and thus can be used as a preventive anti-coagulant and/or as a supplement to existing chemotherapies, producing a double protective effect in COVID-19 infected cancer patients, counteracting the viral- and cancer-induced platelet hyperreactivity [260,261,262].

With regard to *inflammation and multiple organ failure*; treatment approaches of COVID-19 that aim at targeting the viral replication cycle may not be enough for promoting host survival, as it is also necessary to target the virus-induced cytokine release syndrome (CRS) that leads to multiple organ damage and ARDS [263]. Some of the proinflammatory cytokines that are locally and systemically elevated include IL-1β, interferon-γ (IFN-γ), IL-1, IL-6, and monocyte chemoattractant protein 1 (MCP-1). They have been detected in critically ill patients, and they were found to correlate to disease severity [264,265]. Furthermore, upon SARS-CoV-2 entry into the host cell, the disruption of the intracellular environment via ion redistribution results in the activation of NLRP3 inflammasome that increases the secretion of proinflammatory cytokines: IL-1β, IL-18, IL-6 and TNF, further contributing to the imminent cytokine storm and tissue inflammation during respiratory illness [266]. In this context, TQ could have a promising anti-inflammatory role, where it significantly decreases the mRNA expression of the aforementioned proinflammatory cytokines, IL-18, IL-1β and TNF-α, and inhibits IL-6-signaling [16,17,267,268,269]. In cancer patients, inflammasomes are also overexpressed, leading to an increased inflammatory status [270,271]. The disastrous effect of NLRP3 inflammasome in melanoma is owed to its suppression of NK cell activation, necessary for the release of IFN-γ and killing of tumor cells, therefore, ultimately increasing lung metastasis [272,273]. Closely examining the molecular mechanisms of inflammasome complexes in different cancer types revealed that the overexpression of IL-1β causes mobilization of myeloid-derived suppressor cells (MDSCs) and induces gastric cancer [274], while signaling of IL-1 drives the accumulation of MDSCs, promoting primary and metastatic mammary tumors [275]. Moreover, the inflammatory mediator IL-18 is known to accumulate in cancer patients with the ability to fine-tune the activation status of NK cells, depending on the amount of IL-18 [12,276]. It is worth noting that inflammasomes do not always show tumor-promoting effects; in fact, they showed protective roles in some cancer types [277]. Since TQ is an NLRP3 inhibitor, this would consequently decrease the secreted IL-1β, IL-18 and IL-6 and ameliorate pain and inflammation in COVID-19 cancer patients. In addition to NLRP3, eicosanoids play a critical role in the progression of inflammation, fever, allergies, cancer, and pain [206,273,275]. Although the role of eicosanoids in COVID-19 is not well characterized, it is believed that cell debris is able to initiate an eicosanoid and cytokine storm in the context of inflammatory diseases. TQ is also able to target arachidonic acid metabolism [278]. Targeting the upstream portion of the eicosanoid storm will potently inhibit the cytokine storm formation, protecting against inflammation-mediated multiple organ damage in COVID-19 patients.

*Oxidative stress:* oxidative damage is an important aspect in which COVID-19 has a notable impact on cancer patients. The analysis showed that interactions between viral proteins and some of the components of the ROS pathway had been implicated in different cancer types. These interactions may be exploited by SARS-CoV-2 to disrupt the basic processes that modulate mitochondrial respiration, metabolism, and oxidative stress, including certain pathways that are known to be important for the development of tumors [279]. TQ happens to demonstrate potent antioxidant properties, where it significantly attenuates glutathione (GSH) depletion and increases the activity of the glutathione-S-transferase (GST) enzyme. It significantly decreases oxidative stress markers, including thiobarbituric acid reactive substance (TBARS), which is elevated in several cancer types like colon and cervical cancer [280,281]. Furthermore, TQ induces the expression of several detoxifying enzymes, including glutathione reductase, superoxide dismutase 1 (SOD1), catalase, and glutathione peroxidase 2 (GPX) [16,19,20]. The reduction of oxidative stress, therefore, gives a protective effect against multiple organ failure associated with COVID-19 and cancer patients.

*Myocardial injury:* cardiac injury is significantly associated with fatal outcomes in COVID-19. In sepsis, elevated plasma levels of troponin-T (c-Tnt) are an important indicator of cardiac damage. Severe cases of COVID-19 showed elevated plasma levels of c-Tnt and were in higher need of mechanical ventilation compared to those with normal Tnt levels. They also had more frequent malignant arrhythmias and needed glucocorticoid therapy [282]. Increased inflammatory cytokines can directly or indirectly cause cardiac damage, but the precise mechanism of myocardial dysfunction-induced sepsis is still unclear. This is also common in cancer patients, where cardiac injury is a risk factor for mortality and may be indicated by elevated levels of troponin and BNP biomarkers, which could result from either the malignancy or the treatment regimen [13]. For example, certain anticancer medications cause patients to suffer from cardiotoxicity presenting as ischemia or vasospasm. An example of this is CAR-T therapy, which may result in CRS, and proteasome inhibitors, which may cause heart failure [283]. Literature showed that TQ significantly decreased TnT levels and markedly reduced cardiac tissue-inflammatory cell infiltration in the cecal ligation and puncture (CLP)-induced-sepsis group. Additionally, P62 and beclin1 are two major proteins in autophagy, and previous literature showed that P62 expression is increased, while beclin1 is decreased in cardiac damage and in sepsis [284,285,286]. TQ significantly decreased P62 and increased expression of beclin1 in CLP mice, thus decreasing sepsis-induced cardiac damage.

*Lung injury:* COVID-19 causes respiratory symptoms including breathlessness, pneumonia, severe ARDS, lung fibrosis and injury [287]. Studies show that TQ has promising effects in lung protection; it protects against injury and fibrosis induced by LPS, toluene, and cyclo-phosphamide. It also induces the relaxation of the precontracted pulmonary artery [213,214,216,217,218,219,220]. Moreover, TQ inhibits inflammation, neoangiogenesis, and vascular remodeling in various asthmatic models [215,221,222,223]. It also counteracts several processes, including emphysema in air alveoli, activation of hyperplastic lymphoid cells surrounding the bronchioles, and inflammatory cell infiltration [216]. According to Jaber S. Alqahtani et al., 2020, chronic obstructive pulmonary disease (COPD) patients were at a higher risk of more severe COVID-19, especially in smokers, as they were seen to have higher expression of ACE2 in their airways [288,289]. TQ decreases the inflammatory and apoptotic index levels in rats exposed to cigarette smoke and shows anti-inflammatory and cytoprotective effects, as summarized in Table 4 [22].

*Liver injury:* liver injury is a critical, life-threatening COVID-19 complication [290]. It is common in cancer patients, as some chemotherapeutic agents like cyclophosphamide [291,292] and tamoxifen cause hepatotoxicity [293], as reviewed in De La Vega et al. [294]. This demonstrates the potentially fatal ramifications of COVID-19 in cancer patients. TQ shows several promising hepatoprotective effects against toxicities and fibrosis [20,23,24,53,224,226,227,228,230,231,232], where it protects against chemotherapy-induced hepatotoxicity through reducing liver injury and tumor markers expression, hinting atthe use of TQ in the treatment of hepatocellular carcinoma [153,225,229]. Altogether, using TQ provides a dual benefit, where it protects against both COVID-19-induced and chemotherapy-induced hepatotoxicity.

*Kidney injury:* this is another COVID-19 related serious complication as reviewed in [295]. Certain chemotherapeutic regimens make cancer patients more susceptible to nephrotoxicity [296]. TQ shows protective effects in the kidneys through ameliorating oxidative stress and reversing the nephrotoxic effects of various drugs and infections [24,25,226,233,234], as summarized in Table 4. Moreover, TQ shows reno-protective effects in sepsis-induced AKI. Since AKI is majorly mediated by dysregulated activation of inflammasomes and proinflammatory cytokines, the aforementioned anti-inflammatory properties of TQ also appear to be of significant benefit in this context [17], where TQ decreases apoptosis of kidney cells and alleviates AKI. Finally, NFκB is the main transcription regulator of inflammatory genes and has crucial roles in inflammation and the pathophysiology of sepsis. TQ reverses the increased NFκB expression in the kidney from septic mice. Collectively, TQ contributes to the alleviation of sepsis-induced AKI by regulating pyroptosis, proinflammatory cytokines, and apoptosis-related expression [17].

*Neurologic/cognitive manifestations associated with COVID-19:* these manifestations include headache, memory loss, mood changes, vision changes, hearing loss, loss of smell, loss of taste, impaired mobility, limb numbness, tremor, fatigue and myalgia [297]. This is along with cases of encephalitis, necrotizing hemorrhagic encephalopathy, stroke, and epileptic seizures as reviewed in [298]. TQ shows several neuroprotective effects, as summarized in Table 4 [191,236].

TQ and gastrointestinal (GIT) manifestations associated with COVID-19: these include diarrhea, loss of appetite, nausea/vomiting and abdominal pain as reviewed in [299]. TQ shows a promising effect against anaerobic bacteria, thus would alleviate diarrhea. It also acts as a gastroprotective agent, proton pump inhibitor and enhances mucin secretion, which would be additionally beneficial in cancer patients suffering from GIT symptoms [18,26,44,45,238].

*TQ and ocular manifestations and conjunctivitis associated with COVID-19* [300]: literature showed that TQ decreases the symptoms of allergic conjunctivitis nearly as effectively as dexamethasone [239], possibly ameliorating COVID-19 associated conjunctivitis.

Pancreatitis: pancreatic inflammation, along with increased lipases associated with COVID-19, can be reduced with TQ since it has been shown to decrease lipases and protect the pancreas against oxidative stress [16,241].

**Figure 3:**
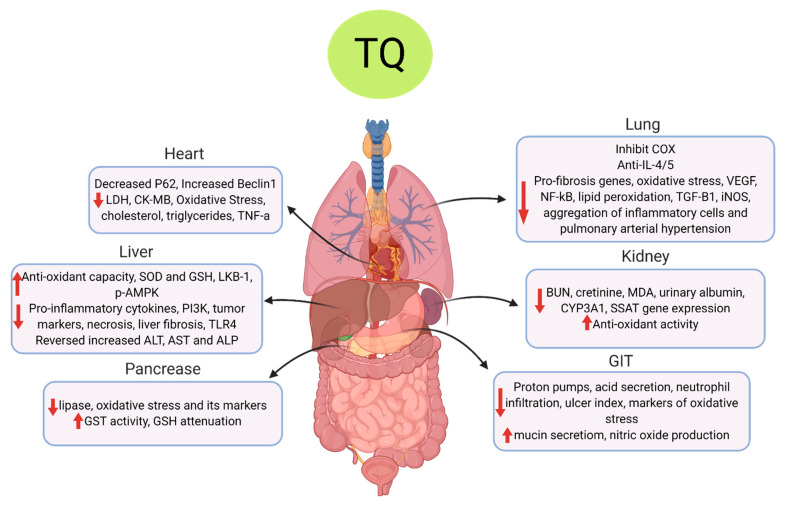
**The multifunctional effects of TQ on COVID-19-associated pathophysiology.** The diagram summarizes the main organ-related and organ protective effects of TQ on the main body organs. In the heart, TQ decreases cardiac enzymes, cholesterol, triglycerides and inflammatory cytokines. In the liver, TQ increases antioxidant enzymes and decreases proinflammatory genes and cytokines, elevated liver enzymes and liver fibrosis. In the pancreas, TQ increases antioxidant capacity by increasing glutathione-S-transferase (GST) enzyme and glutathione (GSH) levels while decreasing lipase and oxidative stress. In the lungs, TQ inhibits cyclooxygenase (COX) enzyme, IL-4/5 levels, NF-ĸB, vascular endothelial growth factor (VEGF), transforming growth factor-β1 (TGF-β1), oxidative stress, inducible nitric oxide synthase(iNOS) enzyme, lipid peroxidation, pro-fibrosis genes and pulmonary arterial hypertension;in the kidney, TQ decreases kidney markers, blood urea nitrogen (BUN), creatinine, malondialdehyde (MDA) and albumin and increases antioxidant activity. In the gastrointestinal tract (GIT), TQ inhibits proton pump, acid secretion, ulcer index, neutrophil infiltration and oxidative stress markers and increases mucin secretion and nitric oxide (NO) production.

Collectively, TQ showed protective effects in the heart, lungs, kidney, liver, GIT, pancreas and alleviated oxidative stress as summarized in Figure 3 and reported in detail in Table 4.

## 7. Conclusions

**Figure 4:**
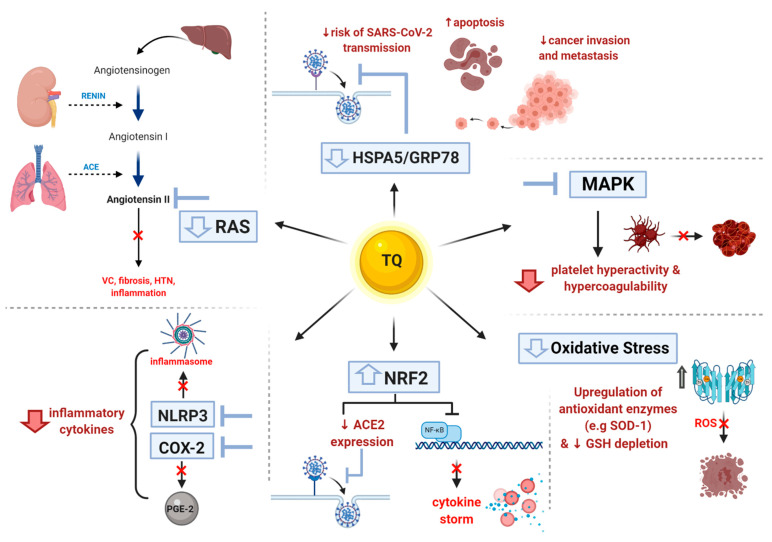
**TQ common effects in SARS-CoV-2 infection and cancer.** The figure summarizes TQ’s actions on molecular targets that cause beneficial outcomes in both SARS-CoV-2 infection and cancer pathogenesis.

The clinical spectrum of COVID-19 can generally range from patients who are paucisymptomatic to those experiencing severe respiratory failure and ARDS, and finally, to those who suffer from systemic manifestations and multiple organ failure. Cancer patients are of particular interest in light of this pandemic; they are more liable to experience a more severe clinical course of the disease, owing to multiple confounding factors, such as increased basal inflammatory state, increased oxidative stress, as well as the very likely presence of existing comorbidities other than the malignancy itself. Amidst the eagerness to vaccinate populations against COVID-19, the aforementioned points still justify the rather meticulous hunt for a drug or an agent that would ideally provide COVID-19 infected cancer patients with double benefits, positively affecting the courses of both their malignancies and the viral infection. In this review, we have hoped to shed light on TQ from *Nigella sativa* seeds and how it has been shown throughout numerous studies to have potential beneficial and protective effects in general and in COVID-19-infected cancer patients in particular, with significantly lower incidences of side effects. First, it reduces the probability of SARS-CoV-2 entry into cells. Second, it helps ameliorate the deleterious effects of CRS in COVID-19-infected cancer patients, thereby protecting against multiple organ damage. Additionally, it is particularly beneficial for cancer patients because it could potentially alleviate multiple chemotherapy-induced toxicities, exhibit anticancer effects, as well as exhibit chemo- and radio-sensitizing effects. Figure 4 summarizes the common molecular targets and effects of TQ that are beneficial in both cancer and COVID-19 patients. Altogether, TQ is a promising solution that may win the battle against COVID-19 and cancer. While TQ is not an FDA approved drug for the treatment of cancer or COVID-19; during the past year, onephase 3 clinical trial was done while threephase 2 clinical trials are currently studying the effect of *Nigella sativa* extracts on immunity and clinical outcomes of COVID-19 patients as per clinicaltrials.gov (Identifier number: NCT04347382, NCT04472585, NCT04401202 and NCT04553705). It is clear that further clinical trials investigating the use of TQ in numerous cancers need to be implemented.

1. Increase in Nrf2 that decreases ACE2 expression lessening the viral entry and inhibits NF-kB that downregulates inflammatory genes and cytokine storm beneficial in viral infection and cancer pathogenesis;
2. Reduction in inflammatory cytokines through NLRP3 inhibition, which in turn inhibits inflammasome formation and all inflammasome components and inhibits COX-2 enzyme hence decreasing inflammatory PGE2 levels;
3. Decrease in RAS activity by attenuating angiotensin II levels (ATII) which abolishes its detrimental effects such as vasoconstriction (VC), fibrosis, hypertension (HTN) and inflammation;
4. Decrease in expression of HSPA5/GRP78, which halts viral entry into the cells and decreases cancer metastasis while enhancing apoptosis.
5. Inhibition of MAPK, which decreases dysregulated platelet activity reducing risks of clotting/coagulopathy.
6. Decrease in oxidative stress by upregulating antioxidant enzymes such as superoxide dismutase (SOD1) and enhancing glutathione (GSH) levels.

## Figures and Tables

**Table 1:** The differential expression of key enzymes essential for viral spike proteins interaction in different cancer types.

Cancer Type	TMPRSS	CTSL
DLBC		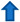
ESCA		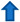
GBM		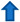
HNSC		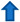
LGG		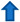
PADD		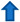
SKCM		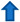
STAD		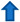
THYM		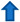
CESC	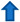	
UCS	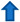	
KICH	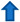	
PRAD	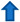	
READ	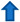	
UCEC	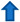	

Abbreviations: TMPRSS, transmembrane serine protease; CTSL; cathepsin L; DLBC, lymphoid neoplasm diffuse large B-cell lymphoma; ESCA, esophageal carcinoma; GBM, glioblastoma multiforme; HNSC, head and neck squamous cell carcinoma; KICH, kidney chromophobe; LGG, brain lower grade glioma; PAAD, pancreatic adenocarcinoma; PRAD, prostate adenocarcinoma; READ, rectum adenocarcinoma; SKCM, skin cutaneous melanoma; STAD, stomach adenocarcinoma; THYM, thymoma; UCEC, uterine corpus endometrial carcinoma [34].

**Table 2:** Thymoquinone (TQ) as a potential adjuvant therapy to minimize SARS-CoV-2(severe acute respiratory syndrome-coronavirus-2) treatments’ side effects.

Drug	Beneficial Effect of Thymoquinone as Adjuvant	Reference
Acetaminophen	-Reversal of acetaminophen-induced lipid peroxide increase in ALT, total nitrate/nitrite, and a decrease in GSH and ATP. -TQ is effective in protecting mice against acetaminophen-induced hepatotoxicity, liver cirrhosis and fibrosis via increased resistance to oxidative and nitrosative stress and decreased serum biomarker enzymes (SGOT, SGPT and ALP)	[23,53]
Diclofenac	-TQ could ameliorate gastrointestinal and renal toxicity induced by high dose diclofenac treatment. -TQ administration, with Diclofenac treatment, alleviated the buildup of poly-unsaturated fatty acids (PUFAs) and diclofenac-induced cell death in kidney cells.	[42,43]
Gentamicin (severe secondary bacterial infections)	-TQ showed a protective role against Gentamicin ototoxicity -TQ significantly prevented the Gentamicin-induced elevations of serum AST, ALT and LDH activities and also tumor necrosis factor-alpha (TNF-α) and total bilirubin levels	[46,47]
Chloroquine	-TQ provides cardio-protective effects by reducing myocardial enzymes following chloroquine induced cardiotoxicity.	[48]
Vancomycin	-TQ reversed the vancomycin induced elevated serum blood urea nitrogen, creatinine and MDA and showed protective effects against injury	[25]
Steroids (methylprednisolone)	-TQ improved oxygenation and protected lung tissue from hazardous effects of human gastric juice (pH 1.2)	[37]
Montelukast	-TQ and montelukast have dose-dependent effects on cilia beat frequency (CBF), extending their mechanism of action in respiratory diseases.	[54]
Ranitidine, H2 blockers, Proton pump inhibitors	-TQ+ ranitidine showed synergistic effects and a more significant decrease in ulcer index. -TQ has novel gastroprotective mechanisms via inhibiting proton pump, acid secretion and neutrophil infiltration while enhancing mucin secretion, and nitric oxide production	[44,45]

Abbreviations: ALT, alanine aminotransferase; AST, aspartate aminotransferase; GSH, glutathione; ALP, alkaline phosphatase; SGOT, serum glutamic oxaloacetic transaminase, SGPT, serum glutamic pyruvic transaminase.

**Table 3:** The chemomodulatory/chemosensitizing and protective effects of thymoquinone in combination with chemotherapeutic agents.

Drugs	Effects	Mechanism	Experimental Model	Reference
Paclitaxel	Chemomodulatory	Downregulating Twist1 Increase apoptotic cell death and autophagy	Breast cancer T47D and MCF-7 cells	[143]
Docetaxel	Chemomodulatory	Blocking PI3K/AKT pathways Enhance Bax, Bid, caspase 3 and inhibiting Bcl-XL	Prostate cancer DU145 and C4-2B cells	[144,145]
Doxorubicin	Chemomodulatory	Upregulate PTEN Inhibiting AKT Increasing Bax/Bcl2 ratio Increase ROS formation	Breast cancer MCF-7 cells	[146,147]
	Chemomodulatory and protective	ROS generation Higher mitochondrial disruption Maintain cardiac myocyte survival	T cell leukemia cells Lymphoblastic leukemia	[148,149,150]
Tamoxifen	Chemomodulatory and protective	Inactivating AKT, XIAP, bcl-xl and Bcl-2 Activating caspase-3, Bax, cytochrome c and p27 Hepatoprotective via antioxidant and anti-inflammatory actions	Breast cancer cells	[151,152,153]
5-fluorouracil	Chemomodulatory	Downregulate Bcl-2, Wnt/β-catenin, NF-kB, Cox2, iNOS, VEGF and PI3k/AKT Upregulate Bax, Bcl-2, TGF-β1 and Samd4	Colorectal cancer stem cells Gastric cancer cells	[154,155,156]
Cisplatin	Chemomodulatory and protective	Block JAK2/STAT3 pathway Inhibition of PI3K/AKT pathway Activating Bax, cytochrome c and caspases Reduced hepatorenal toxicity through antioxidant effect	Esophageal cancer cells Gastric cancer cells Oral squamous cell carcinoma	[138,139,157,158,159,160]

**Table 4:** The beneficial effects of TQ against COVID-19 pathophysiological effects.

COVID-19 Complications	Thymoquinone	References
Cardiac damage	-Protects against hyperlipidemia, cyclophosphamide, doxorubicin and diesel exhaust particle-induced cardiac damage or changes -Counteracts sepsis-induced elevation in p62 and increases beclin-1 expression -Reduced infarct size, improved cardiac function, reduced myocardial enzyme LDH and CK-MB activities and inhibited oxidative stress	[21,48,209,210,211,212]
Lung injury, ARDS, COPD, pulmonary fibrosis	-Inhibited nuclear factor kappa-B (NF-ĸB) in lung tissue -Ameliorates the pathological changes of chronic asthma, inhibits inflammatory changes by antagonizing IL-4/5, and a single dose prevented asthma in the guinea pig model -Showed anti-neoangiogenic effects through inhibition of expression of VEGFR2/PI3K/Akt-signaling pathway -Decreased the inflammatory and apoptotic index levels in rats exposed to cigarette smoke and showed anti-inflammatory and cytoprotective effects against cigarette smoke-induced COPD and LPS induced acute lung injury by COX inhibition -Downregulated pro-fibrosis genes and decreased oxidative stress in lung fibrosis -Counteracted emphysema in air alveoli, lymphoid hyperplastic cells activation surrounding the bronchioles, inflammatory cell infiltration -Ameliorated pulmonary arterial hypertension, Induced relaxation of the precontracted pulmonary artery and caused a decrease in the tension of pulmonary arterial rings precontracted with phenylephrine -Reduced alterations in lungs and inflammatory markers induced by cyclophosphamide and toluene, decreased lipid peroxidation and restored antioxidants -Showed inhibitory effects on the aggregation of inflammatory cells in bronchoalveolar lavage (BAL) fluid and in lung tissues -Significantly decreased serum IgE and showed superior inhibitory effects on iNOS and TGF-β1	[22,213,214,215,216,217,218,219,220,221,222,223]
Liver injury, elevated liver enzymes	-Effectively improved the plasma and liver antioxidant capacity and enhanced the expression of liver antioxidant genes of hypercholesterolemic rats -Protects against oxidative liver damage and ductular proliferation -Reduced liver injury and tumor markers expressions;thus its beneficial in the treatment of hepatocellular carcinoma -Protected against cyclophosphamide, tamoxifen, cypermethrin, cadmium, anti-TB drugs, aflatoxinB1, acetaminophen and paracetamol-induced hepatotoxicity/necrosis/cirrhosis and fibrosis by decreasing the elevated ALT, AST and ALP, enhanced regeneration after tissue damage, decreased oxidative protein damage and increased SOD expression and GSH -Ameliorated liver fibrosis via blocking TLR4 expression and PI3K phosphorylation on the activated hepatic stellate cells -Reduced thioacetamide-induced liver fibrosis and inflammation by decreasing proinflammatory cytokines, inhibiting PI3K phosphorylation and enhanced p-AMPK and liver kinase B (LKB-1)	[20,23,24,53,153,224,225,226,227,228,229,230,231,232]
Kidney damage	-Attenuated oxidative stress and inflammation-reducing renal ischemia-induced damage and several drugs-induced nephrotoxicities -Reversed the vancomycin and doxorubicin-induced nephrotoxicity, reversed elevated serum blood urea nitrogen, creatinine, MDA and urinary albumin excretion -Protected kidney against urinary tract infections (*Escherichia coli*) induced pyelonephritis -Showed protective action against cypermethrin induced shrinkage of glomeruli and necrosis of renal tubules in kidneys (in mice)	[24,25,226,233,234]
Neurological disease, cognitive decline	-Induced a significant increase in expression of neuroprotective proteins such as biliverdin reductase-A, 3-mercaptopyruvate sulfurtransferase, glutaredoxin-3, and mitochondrial Lon protease, a significant decrease in expression of inflammatory cytokines, and NF-κB pathway -Decreased malondialdehyde and nitric oxide metabolites in the brain -Enhanced the thiol content and superoxide dismutase and catalase activities and serum T4 level -Protected against PTU-induced memory impairments in rats -Reversed learning and memory impairments, brain tissue-oxidative damage in hypothyroid juvenile rats and alleviated changes in the hippocampal lipid peroxide level and SOD and AChE activities -Have a protective effect on learning and memory function. It significantly increased the expression of Nrf2, HO-1 proteins and SOD in the hippocampus	[51,235,236,237]
GIT	-Showed significant antimicrobial activity against anaerobic bacteria, thus can be used against diarrhea -Prevented and significantly reduced the appearance of diarrhea and body weight loss in mice and ameliorates dextran sulfate sodium-induced colitis -Inhibited proton pump, acid secretion and neutrophil infiltration, while enhancing mucin secretion, reduces ulcer index, markers of oxidative stress and nitric oxide production -TQ+ ranitidine showed synergistic effects and a more significant decrease in ulcer index -Suppressed spontaneously contracting rabbit jejunum and also relaxed high K induced contractions in the jejunum and guinea-pig ileum, thus can be used in colic and diarrhea	[18,26,44,45,238]
Allergy, allergic conjunctivitis	-Inhibited histamine production -Decreases the symptoms of allergic conjunctivitis almost the same as dexamethasone -Suppressed the Total IgE, OVA-specific IgE and Recruitment of Inflammatory cells -Alleviated allergic inflammation and may be valuable for treating allergic rhinitis	[239,240]
Acute pancreatitis and elevated lipase	-Decreased lipase\amylase ratio -Decreased oxidative stress markers, pancreatic 4-hydroxynonenal (4-HNE), protein carbonyl content and protected pancreatic acinar cells from oxidative stress -Attenuated GSH depletion and increased the activity of GST	[16,241]

Abbreviations: LDH: lactate dehydrogenase; CK-MB: creatininekinase-MB; ARDS: acute respiratory distress syndrome; COPD: chronic obstructive pulmonary disease.

## Data Availability

Not Applicable.
